# Modeling of Shunted Piezoelectrics and Enhancement of Vibration Suppression through an Auxetic Interface

**DOI:** 10.3390/mi14020289

**Published:** 2023-01-22

**Authors:** Maria-Styliani Daraki, Konstantinos Marakakis, Georgios E. Stavroulakis

**Affiliations:** Institute of Computational Mechanics and Optimization (Co.Mec.O), School of Production Engineering and Management, Technical University of Crete, GR-73100 Chania, Greece

**Keywords:** auxetic material, piezoelectrics, shunt circuits, parametric analysis, vibration control

## Abstract

In this study, a new technique is presented for enhancing the vibration suppression of shunted piezoelectrics by using an auxetic composite layer. Finite element models have been created to simulate the dynamic behavior of the piezoelectric composite beam. In particular, 2D FE and 3D FE models have been created by simulating the shunt as a passive controller and their results are compared. Furthermore, a parametric analysis is presented of the circuit elements, i.e., the resistors, inductors, and capacitors and of the auxetic material, i.e., the thickness. It was found that the proposed modification by adding an auxetic layer of a considerable thickness enhances the electromechanical coupling and indirectly influences the vibration control of the whole structure. However, the use of 3D modeling is necessary to study this auxetic enhancement.

## 1. Introduction

The vibration damping of the structures due to an external excitation is one of the most common problems in engineering. A reduction in the vibration is needed for functional reasons, for example, for tracking the accuracy in flexible robotics, which leads, amongst other things, to reduced fatigue which, in this way, extends the life of the vibrating components. Several techniques have been proposed on this subject in the literature [1,2]. Composite layered structures and microstructures with bonded piezoelectric materials can be used for this purpose [3,4].

Piezoelectric materials are distinguished by their special characteristics. Specifically, during the application of a mechanical strain, opposite electrical charges are generated on opposite crystal surfaces, which are analogous to the magnitude of the mechanical strain. This phenomenon is called the direct piezoelectric effect. However, when an electric field is applied to the material, then mechanical deformation is generated. This phenomenon is called the inverse piezoelectric effect. The piezoelectric effect was discovered by the brothers Jacques and Pierre Curie in the late nineteenth century [5]. Additionally, piezoelectric materials are widely used for vibration damping, energy harvesting, and many other applications due to their excellent electromechanical coupling properties and their frequency response. Piezoelectric transducers can be found in several different shapes, the most common of which are thin sheets known as piezoelectric patches [6]. In addition, shunt circuits, which are paired with piezoelectric elements, form a structural, usually passive, damping method. This method is called shunt damping and it has attracted a lot of scientific interest during the past two decades in vibration control engineering due to its simplicity and applicability in real life applications. Among others, this method can be used in civil structures [7], in smart panels for noise reduction [8], in the design of hard disk drives [9], in the damping of turbine blades [10], etc. 

Shunt circuits have also been used for the development of smart metamaterials in order to enhance their properties. For example, in [11], synthetic impedance shunt circuits were used for the design and analysis of piezoelectric metamaterial beams. In [12], the metamaterial enhancement is studied using periodic piezoelectric arrays and shunt circuits. This method is based on impedance and it aims to construct a parametric design of the electrical elements for mitigating wave propagation and vibration attenuation. 

Auxetic materials constitute a kind of smart metamaterial with a negative Poisson’s ratio. As opposed to conventional materials, the auxetic materials expand laterally under tension and contract under the action of compressive forces. This abnormal behavior is generally achieved through the geometric modifications in the mechanical metamaterial which are based on micromechanical cellular deformation and, thus, different geometries can be proposed which help demonstrate this “auxetic feature”. Microstructures, such as star-shaped ones [13] or chiral ones [14], lead to auxetic behavior. Auxetics, due to strain-inversion, are used for the enhancement of the performance in piezoelectric sensors such as hydrophones [15]. According to [16], in recent years, with the emergence of mechanical metamaterials and additive manufacturing, the auxetic materials have obtained an important role. They have been introduced in order to enhance the output power and the efficiency of the piezoelectric energy harvesters. These materials have witnessed several engineering applications ranging from impact and shock absorption to sound absorption. 

The combined usage of the two aforementioned techniques is presented here by adding an auxetic composite layer to the piezoelectric shunt patches. Piezoelectric materials are used for the modification of their structural characteristics in order to achieve a better attenuation, such as the one in [3]. The objective of the present study is to enhance the damping performance of the whole structure by adding suitably designed shunted piezoelectrics. For this study, simplified homemade two-dimensional and Multiphysics two- and three-dimensional models have been created. Optimization is used for the choice of the parameters of the electric shunted circuit. The results of the various models are comparable and validated with the published results. The effectiveness of the auxetic layer to the vibration suppression of the shunted piezoelectric is demonstrated. Only the 3D model is able to show this effect. Although the ability of auxetics to enhance the effectiveness of the sensors has been presented in the literature, an application on shunted vibration suppression systems has not been considered. This is a novel contribution of the present work. The reduction in the vibration depends on the thickness of the auxetic layer. In this first investigation, a homogenized auxetic layer is considered, without entering into the design of a possible microstructure that leads to a negative Poisson ration and could provide optimal results.

The present paper is divided intο five parts. After the present introduction, Section 2 refers to the theoretical part of the shunt circuits. The following section is dedicated to the description and usage of auxetic materials. The numerical results of the investigation are presented next, while the final section is devoted to the conclusions of the study.

## 2. Shunted Piezoelectric Circuits

A shunted piezoelectric circuit consists of a piezoelectric transducer which is connected (shunted) to an electric impedance, such as a resistance, an inductance, a capacitance, or a suitable combination of them [17], and is usually bonded on smart structures such as beams. Different types of passive shunt circuits are presented based on any possible combination of the electrical components. Such systems can be categorized into two major groups, linear and non-linear circuits, as described in [17]. Another categorization can be divided into passive and active circuits. The nonlinear shunts usually involve switches and/or systems in a variable resistance. A schematic of shunt circuits is summarized along with some common control principles and the equations of these systems are summarized in the review article of [18]. Τhe purpose of shunt damping is the suppression of the vibrations of the structures at several frequencies. In the case of the resonant shunt, which consists of a resistor R and an inductor L either in the series or in parallel, the objective is to suitably tune these parameters of the circuit in order to achieve the maximum attenuation of the selected frequency. These systems are also called single mode systems as they are tuned to attenuate one specific eigenmode [17]. Further advanced methods have been proposed, in the case of the control of more eigenfrequencies, known as multiple modes, such as multi-mode shunts [19]. Then, multimode vibration control can be achieved by using piezoelectric transducers shunted with a multiterminal network, as described in [2]. There are special categories of multi-mode shunt circuits for a reduction in the vibration which have attracted huge attention from the research community in the past few years and their significance is attributed to the suitable tuning of the values of the electric parameters, which are the resistance, the inductance, and the capacitance. The basic principles of passive damping using shunts are given analytically in the book of Preumont [20]. More information about the shunt piezoelectric circuits and their applications to control noise and vibration control can be found in the review article [21].

The modeling of the piezoelectric shunt system follows the methodology outlined, for example, in Jeon, 2009 [22]. In general, the electromechanical coupling introduced by piezoelectric materials is described accordingly on the IEEE Standard on Piezoelectricity, 1987 [23], as:

(1) $$\left\{\begin{array}{c} T\\ D \end{array}\right\}=\left[\begin{array}{cc} c^{E} & -e\\ e^{t} & \varepsilon^{s} \end{array}\right]\left\{\begin{array}{c} S\\ E \end{array}\right\}$$

where

*t*: matrix transpose.
*T:* stress vector [N/m^2^].
*D:* electric displacement vector [C/m^2^].
*S:* strain vector.
*E:* electric field vector [V/m].
*c^E^:* elasticity stiffness matrix calculated evaluated at constant electric field.
*e:* piezoelectric stress matrix.
ε*^S^*: dielectric matrix evaluated at a constant mechanical strain.

Using Hamilton’s principle and finite element discretization in the coupled electromechanical system on a given structure, the coupled matrix equations can be written as follows:

(2) $$\left[\begin{array}{cc} M & 0\\ 0 & 0 \end{array}\right]\left\{\begin{array}{c} \ddot{w}\\ \ddot{v} \end{array}\right\}+\left[\begin{array}{cc} C & 0\\ 0 & 0 \end{array}\right]\left\{\begin{array}{c} \dot{w}\\ \dot{v} \end{array}\right\}+\left[\begin{array}{cc} K & \Theta \\ \Theta^{t} & -C_{p} \end{array}\right]\left\{\begin{array}{c} w\\ v \end{array}\right\}=\left\{\begin{array}{c} F\\ q \end{array}\right\}$$

where [M], [C], [K], and [Θ] represent the global mass matrix, the global damping matrix, the global stiffness matrix, and the electromechanical coupling matrix of the host structure and the piezoelectric material, respectively, [$C_{p}$] is the inherent piezoelectric capacitance matrix, {F} is the applied mechanical force vector, {q} is the electric charge vector, {w} is the generalized mechanical coordinate, and $\left\{v\right\}$ is the generalized electrical coordinate, which is the physical voltage at the piezoelectric electrodes. The damping matrix [C] is assumed to be a proportional viscous damping. 

From Equation (2), the equation of the motion of the piezoelectrically coupled electromechanical system consists of two sets of coupled equations:

(3) $$\left[M]\{\ddot{w}\}+[C]\{\dot{w}\}+[K]\{w\}+[\Theta ]\{v\}=\{F\right\}$$

(4) $${\left[\Theta \right]}^{t}\left\{w\right\}-\left[C_{p}\right]\left\{v\right\}=\left\{q\right\}$$

From these two sets of coupled Equations (3) and (4), the first set describes the equilibrium condition of the mechanical forces and the second set is an electrodynamics condition of the electric potential. These two sets of equations can be employed to derive the piezoelectric passive damping force by means of shunt damping circuits connected to the piezoelectric electrodes. In more detail, after obtaining the appropriate design of the electric part, the solution of Equation (4) for v and the combination of the find part (3) leads to the description of the system in the form of a modified mechanical system that includes the effect of the shunted circuit.

When a piezoelectric patch is shunted by an impedance $Z_{sh}$, the shunt damping voltage across the shunt damping network can be represented by the current–voltage relationship in the Laplace domain as:

(5) $$V_{sh}\left(s\right)=Z_{sh}(s)\cdot I_{sh}(s)$$

where $V_{sh}\left(s\right)$ is the voltage across the impedance and $I_{sh}(s)$ is the current flowing through the impedance. The current can be also obtained by differentiating Equation (4). By substituting it into Equation (5), then:

(6) $$V_{sh}\left(s\right)=Z_{sh}\left(s\right)\cdot \dot{q}\left(s\right)=Z_{sh}\left(s\right)\cdot {({\left[\Theta \right]}^{t}\left\{w\right\}s-\left[C_{p}\right]V}_{sh}\left(s\right)s)$$

and rearranging it, the shunt voltage equation is derived as follows:

(7) $$V_{sh}\left(s\right)=\frac{{Ζ}_{sh}(s){\left[\Theta \right]}^{t}\left\{w\right\}s}{1+{Ζ}_{sh}(s)\left[C_{p}\right]s}$$

where *s* is the Laplace operator.

Substituting Equation (7) into Equation (3), the governing equation of the shunted piezoelectric can be derived by considering the additional passive piezoelectric damping force as follows:

(8) $$\left[M\right]\left\{\ddot{w}\right\}+\left(\left[C\right]+Z_{total}\left[\Theta \right]{\left[\Theta \right]}^{t}\right)\left\{\dot{w}\right\}+\left[K\right]\left\{w\right\}=\left\{F\right\}$$

where the total electrical impedance of the shunted piezoelectric $Z_{total}$ includes the inherent capacitance of the piezoelectric and can be expressed by:

(9) $$Z_{total}=\frac{Z_{sh}}{1+Z_{sh}\left[C_{p}\right]s}$$

Tuning rules for piezoelectric shunts aiming to mitigate single and multiple structural resonances are presented in [22,24], respectively.

The various models, which are used in this paper, are described in this section. More specifically, they are categorized on reduced 2-D and full 2-D or 3-D Multiphysics models and are outlined below. 

### 2.1. 2-D Simplified Model

**Figure 1:**
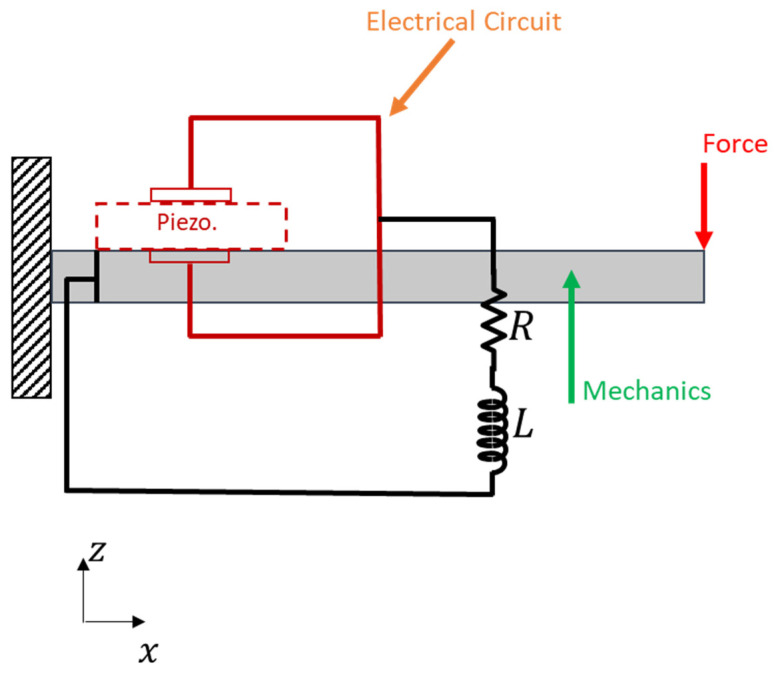
Mechanical model, interface to electrical circuit through a virtual PZT interface.

In this model, as can be seen in Figure 1, which is simulated in a MATLAB environment, the host beam is discretized using finite elements. The piezoelectric phenomenon acts only as an interface to transform the mechanical signal into an electrical one, which is subsequently fed into the electrical circuit. The advantage of this modeling process is the economy of recourses that is mentioned in the work of our team [25]. 

### 2.2. 2-D or 3-D Multiphysics Model

**Figure 2:**
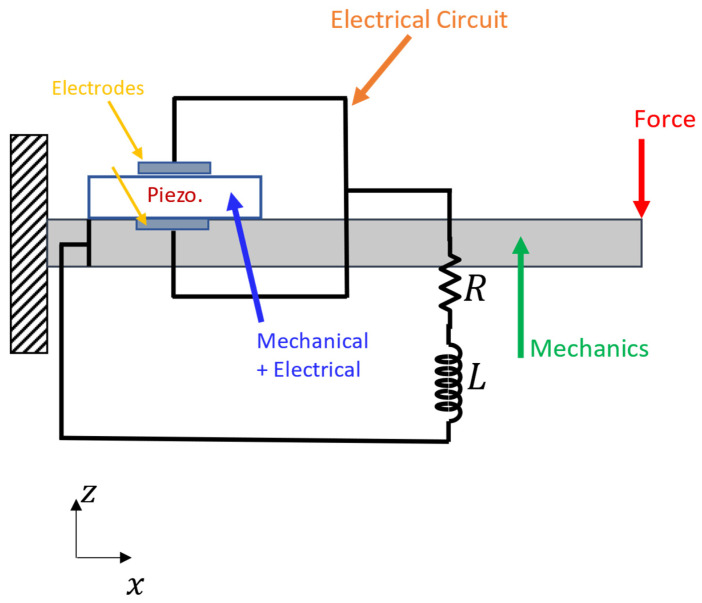
Mechanical and electrical model work together (Multiphysics model).

On the other hand, in a Multiphysics modeling environment, i.e., COMSOL Multiphysics, mechanical and electrical effects appear simultaneously, as can be seen in Figure 2. In this way, the piezoelectric patches are much better simulated and the electrodes can be placed in a more precise way. This model can describe in a more detailed way one of the problems at hand, which is, among others, the effect of the auxetic microstructure. The electric circuit is modeled similarly in two approaches.

## 3. Auxetic Materials

**Figure 3:**
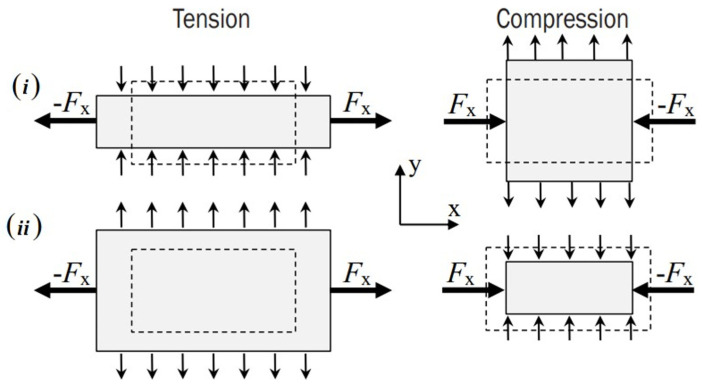
Mechanical behavior applying a tensile and compressive load—(**i**) non-auxetic material and (**ii**) auxetic material. Reproduced from source [27] under CC-BY licence.

As the most studied branch of mechanical metamaterials, auxetic materials exhibit counterintuitive behavior during deformation. To be more specific, under uniaxial compression (tension), conventional materials expand (contract) in the directions which are orthogonal to the applied load. In contrast, auxetic materials become wider when stretched and thinner when compressed. Thus, they have the exact opposite behavior in comparison to the conventional materials. These materials have a negative Poisson’s ratio, the material property which provides information about the modification of the length perpendicular to the loading direction. In the bibliography, these are also referred to as auxetics. In such materials, this special characteristic is usually caused by artificial hinges which appear inside the material’s microstructure. The non-auxetic and auxetic behavior during loading is schematically depicted in Figure 3. Further information about the auxetic behavior of the materials can be found in [3,26,27,28].

In particular, a thin layer with an in-plane negative Poisson’s ratio expands in all directions if pulled towards one of them. Let us recall that the upper side of a three-dimensional beam, when bending, expands in the longitudinal direction of the beam and shrinks in the perpendicular direction along the thickness of the beam. Therefore, an isotropic piezoelectric patch is not used effectively since a positive Poisson coefficient of the host beam’s structure prevents its expansion in both directions. An auxetic layer of a considerable thickness between the host’s structure and the piezoelectric patch transforms the deformation from perpendicular to the loading direction and allows for a full exploitation of the electromechanical coupling. This property has been considered for the enhancement of energy harvesting based on piezoelectric materials; see, among others [29,30,31]. This effect is exploited in the present paper.

The proposed method of using an auxetic layer in order to transform a tensile–compressive input at the upper surface of a bending beam into a tensile–tensile signal at the piezoelectric patch may have a broader applicability. In fact, the usage of sensors for measuring the information coming from several directions is an essential element in smart skin, wearable electronics and soft robotics applications. Furthermore, the technology may allow for the usage of different smart materials, for example, piezoresistives or smart gels. For more information, the reader may consult the recent literature in these topics [32,33,34].

The enhancement of the shunted piezoelectric vibration suppression is proposed here. The proposal of using an auxetic layer is quite general; it is based on the homogenized properties of the layer and does not include the details defining the kind and other quantities of the microstructure that lead to the auxetic behavior.

**Figure 4:**
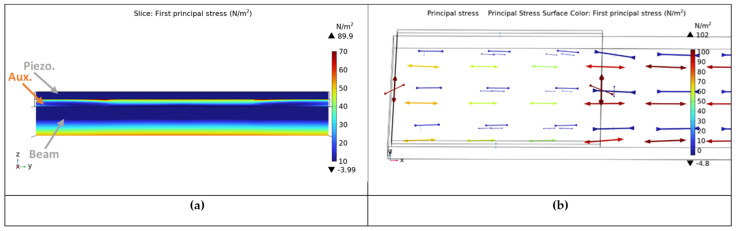
Stresses for the auxetic layer. (**a**) cross-section and (**b**) principal stresses.

**Figure 5:**
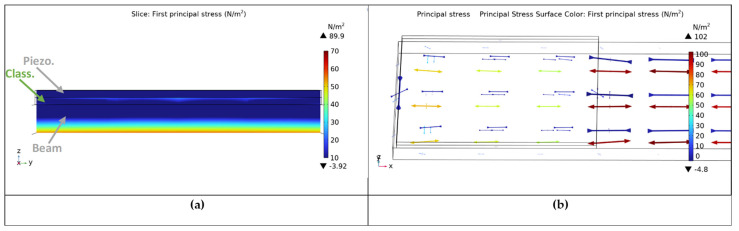
Stresses for the classical layer. (**a**) cross-section and (**b**) principal stresses.

In order to demonstrate the function of the auxetic layer, plots of the stresses within the patch for a positive and negative Poisson’s ratio are compared, and this explains the function of the previously described mechanism; see the stress distribution within a cross-section of the beam in Figure 4 and Figure 5, respectively. The different stress distribution is due to the ability of the auxetic layer to transform a tension–compression signal from the surface of the host beam to a tension–tension one on the surface of the piezoelectric patch. It must be noted that the usage of 3D modeling is necessary for the study of this effect, which is invisible in 2D models.

## 4. Numerical Results

The present section is organized into three parts. First, the same structure has been modelled by the two previously described methods. Then, a parametric investigation of the circuit values is presented. Finally, an auxetic enhancement layer is added and its influence on the response of the structure is investigated.

### 4.1. Simulation Models and Comparison

In the present investigation, the structure which has been considered is the one used in References [5,35]. The host beam is made of aluminum and the two piezoceramics of type PIC151 are placed 0.5 mm away from the fixed end. The piezoelectric patches are symmetrically attached at the top and the bottom surfaces of the host’s structure. The material and geometric properties of the structure are given in Table 1. The proposed FE model was used for the determination of the frequency response and the harmonic force was applied at the tip of the beam. The system response was determined at the same position. A concentrated mass of 4.2 g is added at the tip of the beam to model the magnet used in the non-contact electromagnetic driving system.

In Table 1, the geometric and material parameters and the R-L circuit are presented. The results of a reduced-order 2D and a Multiphysics 3D model have been compared and have been validated with the published results. From the mechanical boundary conditions aspect, a cantilever beam is fixedly supported at one end and freed at the other end, where a harmonic load is applied. For the electric model, the boundary conditions have been defined in a different way in each software. On MATLAB, the formulation is based on Timoshenko beam theory. On COMSOL, it is necessary to determine the parameters for the electric model and the circuit function, i.e., the Zero Charge node which is the default boundary condition.

The finite element formulation which is used by the 2D home-made program is based on the super-convergent FE approach developed by Foutsitzi et al. [25]. For information regarding the mesh element quality of the reduced-order 2D model, the mesh consists of 41 one-dimensional beam elements. Similarly, the mesh of the Multiphysics 3D model consists of 468 quadrilateral elements. The optimization of the shunted parameters was carried out in a MATLAB environment. The particle swarm optimization (PSO) method was applied and the optimal solution was obtained. The parameters of the optimization algorithm are given in detail in Table 2.

In addition, the optimal values of the R-L circuit are presented along with the system response (FRFs) graph from each software. In the beginning, the optimal values of the resistance R and the inductance L of a single-mode resonant shunt circuit, in order to suppress the vibrations of a similar beam structure around the second eigenfrequency, are given in Table 3 and Table 4.

**Figure 6:**
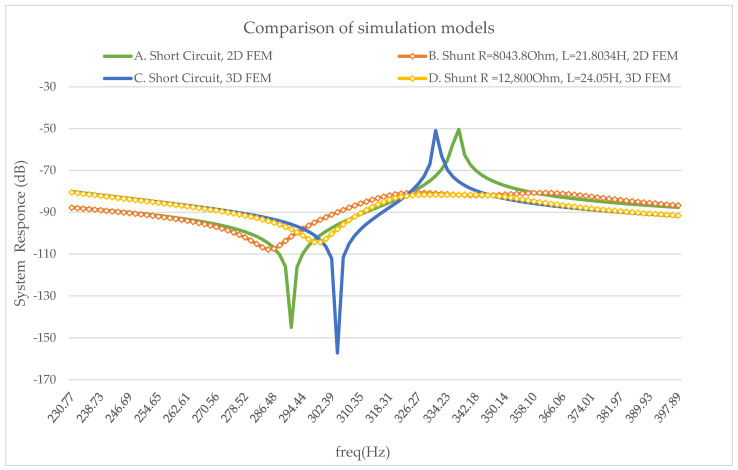
Simulated frequency response of the two models. Aluminum beam, Piezo. PIC151 and Optimal RL circuit values. A. Short circuit 2D FEM MATLAB, B. Shunt circuit R = 8043.8 Ohm L = 21.8034H 2D FEM MATLAB, C. Short circuit 3D FEM, D. Shunt circuit R = 12,800 Ohm L = 24.05 H 3D FEM.

The damping effect of the shunt circuit around the second eigenfrequency, from both models, is depicted in Figure 6. The results from this comparison have a good agreement. So, the correctness of the models is validated.

The technology for damping more than one eigenfrequency has been evaluated by our research team and has been found to be operating effectively. The challenge was the damping of more than one eigenfrequency. In the literature, these kinds of circuits are called multimode shunt damping circuits. More details are given in [5,35,36].

### 4.2. Parametric Investigation of the R-L Circuit Elements

The investigation of the electric circuit parameters, i.e., the resistors, inductors, and capacitors, is presented. The motivation for this investigation comes from the studies of Lossouarn B. [37] and Daraki M.-S. [5]. The structure follows the model of Paragraph 4.1.

**Figure 7:**
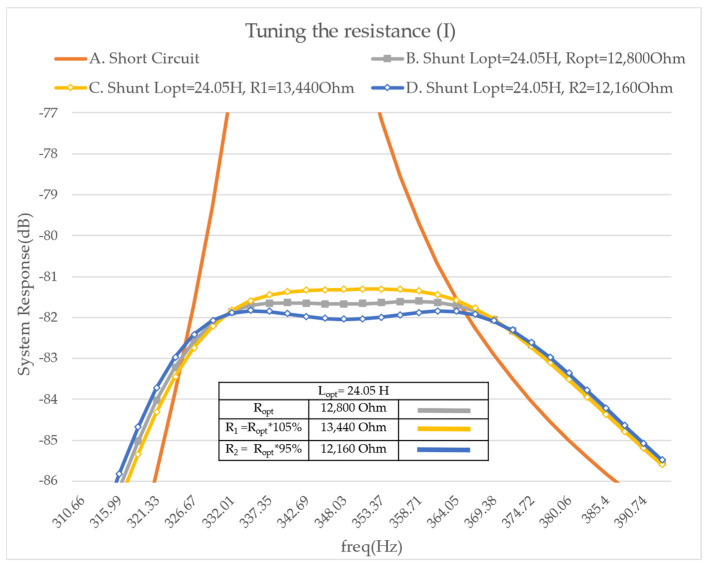
FRF Modulus on mode 2—tuning the resistance— A. with a short circuit, B. with the optimal resistance, C. with twice the optimal resistance, D. with half of the optimal resistance.

First, resistance is added into the shunt in order to obtain a vibration reduction over a broader frequency range. Based on the literature, the optimal resistance leads to a significant reduction in the vibration when compared to the cases involving a short-circuited PZT or a purely inductive shunt. Then, the optimal tuning of the resonant shunt can be validated. The influence of the resistance R is illustrated in Figure 7 when the inductance L is tuned to its optimal value. It is noticed that if one doubles the value of optimal resistance two local maxima appear, whose value increases with the increase in the resistance. On the contrary, half of the optimal resistance leads to a local minimum surrounded by two local maxima that offer approximately the same value. Then, the optimal tuning then corresponds to a case where two maxima appear, but their amplitude is minimized. In the following chart (Figure 7), this optimal damping corresponds to the limit between the so-called under- and overdamped cases.

**Figure 8:**
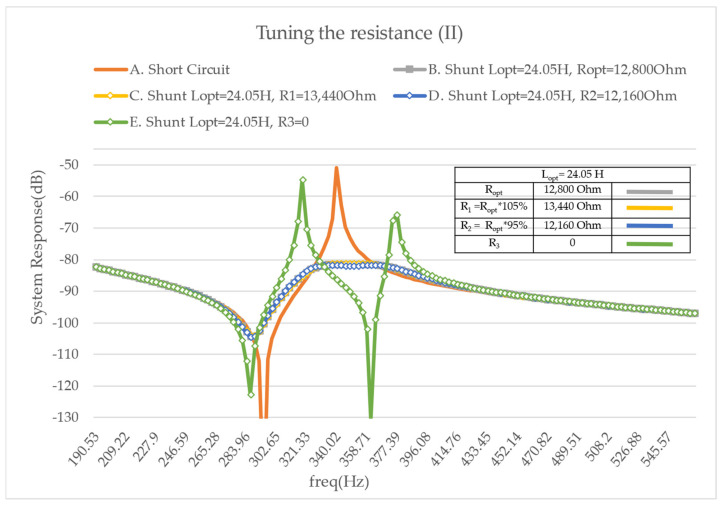
FRF Modulus on mode 2—tuning the resistance— A. with a short circuit, B. with the optimal resistance and inductance, C. with twice the optimal resistance, D. with half of the optimal resistance, E. with the optimal inductance and no resistance. The short circuit and under—, optimal—, and overdamped cases are repeated from Figure 7 for competence.

Additionally, Figure 8 shows that the use of a resonant shunt without any resistance creates antiresonance behavior when the inductance is tuned to its optimal value. In particular, it is the same case as before with an extra electric condition.

**Figure 9:**
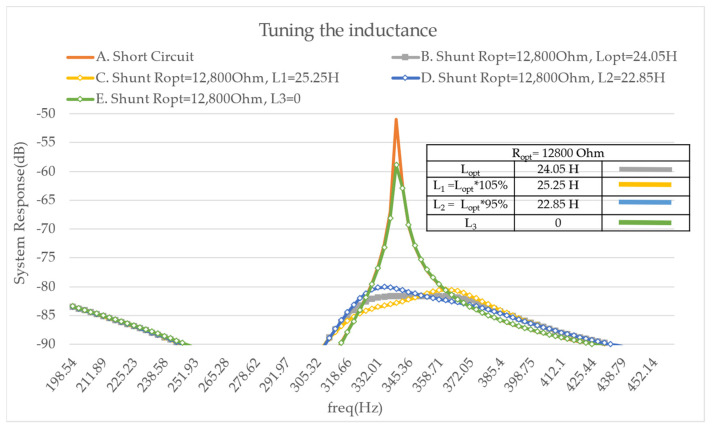
FRF Modulus on mode 2—tuning the inductance—A. with a short circuit, B. with the optimal resistance and inductance, C. with 105% of the optimal inductance, D. with 95% of the optimal inductance, E. with the optimal resistance and no inductance.

Furthermore, the tuning of the inductance is shown in Figure 9, where the resistance is set to its optimal value. According to the literature, the use of an inductance value that differs from the mathematic equation increases the amplitude of one of the two maxima. Thus, the different values of the inductance are investigated and this effect can be observed in Figure 9.

### 4.3. Investigation of Auxetic Materials and Results

In the last part of this section, a method for enhancing the vibration suppression is proposed. The beam is equipped with a PZT patch and an auxetic composite layer is placed between the PZT patch and the host’s structure. It should be noticed that an auxetic enhancement can only be studied by using 3D models. The model of Paragraph 4.1 with only one piezoelectric patch of type PIC 151 is used (see Table 5).

In the next table, Table 6 and Table 7 provide more details for the auxetic composite layer.

Moreover, the parameter of interest is the thickness of the auxetic composite layer. In the literature, it is known that the existence of the auxetic improves the damping of the vibrations on the target’s structure. In this study, this parameter has been found to influence the effectiveness of damping. Specifically, three values of thickness have been considered.

(1) hA= 0.25 mm (hA = hp/2), hA < hp.
(2) hA = hp = 0.5 mm.
(3) hA= 0.7 mm, hA > hp.

The results are summarized in Table 8, where the difference in the various thicknesses is visible.

**Figure 10:**
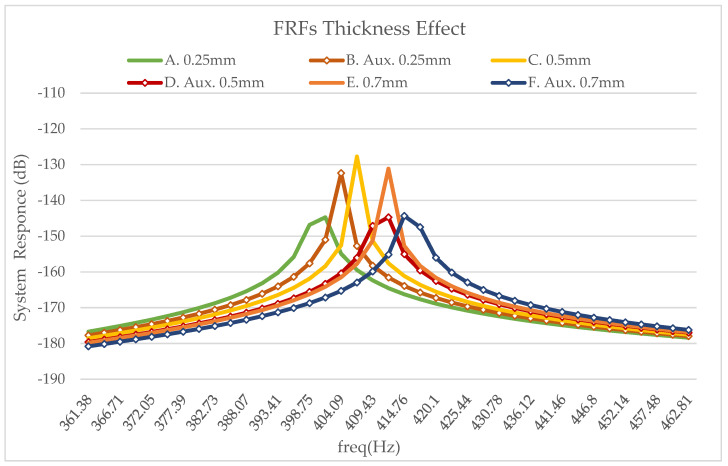
Simulated frequency response for the second eigenmode. Aluminum beam, Piezo. PIC151, additional layer hA and open circuit- A. vxy = 0.202 hA = 0.25 mm, B. vxy = —0.202 hA = 0.25 mm, C. vxy = 0.202 hA = 0.5 mm, D. vxy = —0.202 hA = 0.5 mm, E. vxy = 0.202 hA = 0.7 mm, F. vxy = —0.202 hA = 0.7 mm.

For each case, the magnitude of the frequency response of the tip displacement before shunt damping is depicted. Namely, the circuit is open and each FRF graph shows the result of a separate study. The result curves refer to conventional and auxetic materials, respectively, and they are presented in Figure 10.

The following conclusions can be drawn from the presented parametric investigation. The enhancement of the vibration suppression is considerable when the thickness of the auxetic layer is equal to the thickness of the piezoelectric patch. When the thickness of the auxetic is higher than the thickness of the piezoelectric patch, there is still a reduction but a relatively low one in comparison with the optimal value. On the contrary, in the first case, the one with a very thin auxetic layer, one observes that there is no enhancement at all. Obviously, the thickness of the layer is not sufficient to allow for the development of the beneficial auxetic enhancement.

Considering the ideal thickness of the auxetic layer, hA= 0.5 mm, a R-L resonant shunt piezoelectric circuit has been added. The results demonstrate that the addition of an auxetic layer improves the efficiency of the shunt system. The two previously used materials have been studied for comparison reasons. In each case, the optimal values of the R-L circuit have been calculated. For a classical layer between the host beam and the piezoelectric patch, the optimal values are R= 2800 Ohm and L= 9.45 H, while for an auxetic layer, the best solution is R= 2800 Ohm and L= 8.7 H. The resistance value was the same in both cases.

**Figure 11:**
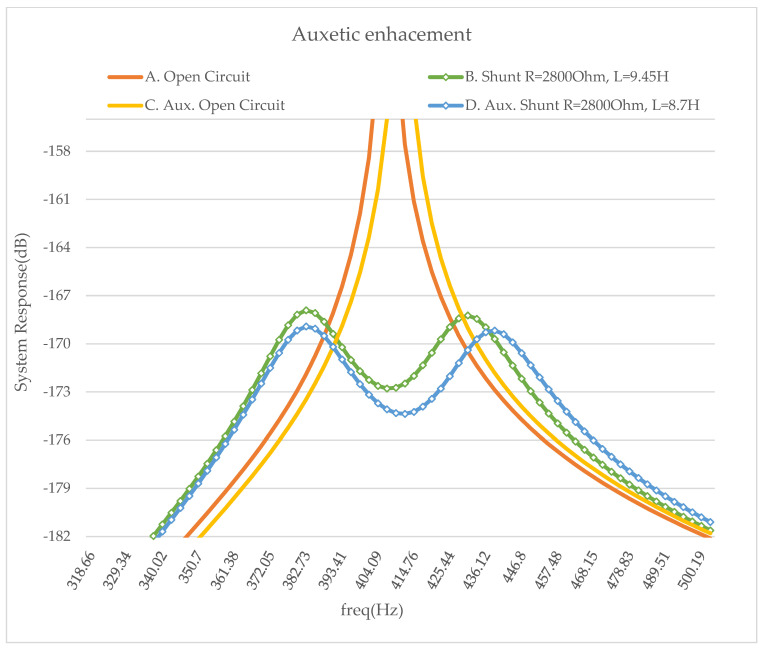
Simulated frequency response of the model. Aluminum beam, Piezo. PIC151, additional layer hA = 0.5 mm and Optimal RL circuit values- A. Open circuit vxy > 0, B. Shunt circuit R = 2800 Ohm L = 9.45 H vxy > 0, C. Open circuit vxy < 0, D. Shunt circuit R = 2800 Ohm L = 8.7 H vxy < 0.

In Figure 11, it is clear that the auxetic (blue line) enhances the damping of the whole structure.

## 5. Conclusions

In this article, a finite element formulation of the coupled electromechanical problem of an elastic structure equipped with piezoelectric patches has been proposed. For comparison, two different computational models have been created, a two-dimensional and a three-dimensional one, based on Multiphysics general purpose software. Furthermore, a parametric analysis of a resonant circuit has been performed which demonstrates the effect of circuit elements in the suppression of a vibration. Moreover, the influence of an auxetic enhancement layer on the structural response is investigated.

From the above analysis, it is necessary to take into consideration that only the optimal values of the R, L, and C parameters are suitable for a maximum attenuation. The optimal values of the parameters can be calculated from the mathematical equations in [39]. The efficiency of the proposed damping methods was indicated by the perfect agreement with the theoretical and experimental published results [40]. These results underly the importance of optimization for the fine tuning of the shunt piezoelectric systems.

It is also worth mentioning that an auxetic layer enhances the shunt damping effect. A homogenized auxetic layer with a sufficient thickness is required based on the presented numerical experiments. Further flexibility can be added to the system by using adapted shunted piezoelectrics or through the modification of the circuit assembly. In addition, the functionally graded design of the auxetic layer is possible by using topology optimization. These topics will be investigated by our group in the future.

## Figures and Tables

**Table 1:** Geometric and material parameters of the compound structure.

Parameters	Beam	Piezo.
Length *L* (mm)	170	25
Width b (mm)	20	20
Thickness h (mm)	2	0.5
Young’s modulus E (GPa)	72	66.7
Shear modulus $G_{12}$ (GPa)	27.48	25.46
Poisson’s ratio, $v_{12}$	0.31	0.31
Density, $\rho_{}(\mathrm{Kg}/m^{3})$	2800	8500
Piezoelectric constant ${\tilde{e}}_{31}^{}$ ($C/m^{2})$	-	−14
Dielectric constant ${\overset{-}{\xi }}_{33}^{}$ (nF/m)	-	2068$\varepsilon_{0}$

$\varepsilon_{0}=8.854\times 10^{-12}$ F/m is the free space permittivity.

**Table 2:** Numerical values for the parameters of the optimization algorithms.

Parameters of PSO	Numerical Values	
Design variables	**R**	**L**
Lower bound	0	0
Upper bound	10,000	200
Number of iterations	50	
Population	25	
Inertia	1	
Inertia weight damping	0.99	
Acceleration factors	c1 = 1.5	c2 = 2

**Table 3:** Optimal shunt element values based on a 2D FEM model.

Element Values of RL Circuit—Aluminum PIC 151, PSO MATLAB	
Parameters	Values
Resistor R (Ohm)	8043.8
Inductor L (H)	21.8034

**Table 4:** Optimal shunt element values based on a 3D FEM model.

Element Values of RL Circuit—Aluminum PIC 151, FEM 3D	
Parameters	Values
Resistor R (Ohm)	12,800
Inductor L (H)	24.05

**Table 5:** Numerical values of system parameters.

	Aluminum Beam		PZT: PIC 151		Auxetic Composite Layer	
Parameters	Beam	Values	Piezo.	Values	Auxetic	Values
Length (mm)	lb	170	lp	25	lA	25
Thickness (mm)	hb	2	hp	0.5	hA	0.5
Width (mm)	b	20	b	20	b	20
Patch position (mm)			x_	0.5	x_	0.5
Density (kg/m^3^)	rb	2800	rp	8500	rA	80
Young’s modulus (GPa)	Yb	72	Yp	66.7		
Poisson’s ratio	vb	0.31	vp	0.34		
Piezoelectric constant (C/m^2^)			${\tilde{e}}_{31}^{}$	−14		
Dielectric constant (F/m)			${\overset{-}{\xi }}_{33}^{}$	2068$\varepsilon_{0}$		

$\varepsilon_{0}=8.854\times 10^{-12}$ F/m is the free space permittivity.

**Table 6:** Numerical values of system parameters.

Parameters	Auxetic	Values
Length (mm)	lA	25
Thickness (mm)	hA	0.5
Width (mm)	b	20
Patch position (mm)	x_	0.5
Density(kg/m^3^)	rA	80

**Table 7:** Mechanical properties have been taken from [38].

Parameters	Auxetic	Values
Young’s modulus (MPa)	E_x_	10,352
Young’s modulus (MPa)	E_y_	177,236
Young’s modulus (MPa)	E_z_	10,352
Poisson’s ratio	v_yx_	−0.202
Poisson’s ratio	v_yz_	0.202
Poisson’s ratio	v_xz_	0.430
Shear modulus (MPa)	G_yx_	4115
Shear modulus (MPa)	G_yz_	4115
Shear modulus (MPa)	G_xz_	3620

**Table 8:** Auxetic Composite Layer—Thickness Effect.

hA = 0.25 mm < hp	** 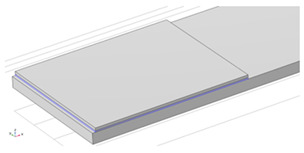 **
hA = hp = 0.50 mm	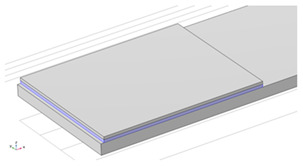
hA = 0.7 mm > hp	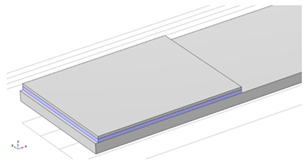

## Data Availability

Models can be provided after reasonable request.
